# Community Knowledge, Health Beliefs, Practices and Experiences Related to Dengue Fever and Its Association with IgG Seropositivity

**DOI:** 10.1371/journal.pntd.0002789

**Published:** 2014-05-22

**Authors:** Li Ping Wong, Sazaly AbuBakar, Karuthan Chinna

**Affiliations:** 1 Department of Social and Preventive Medicine, Faculty of Medicine, University of Malaya, Kuala Lumpur, Malaysia; 2 Julius Centre University of Malaya (JCUM), University of Malaya, Kuala Lumpur, Malaysia; 3 Department of Medical Microbiology, Faculty of Medicine, University of Malaya, Kuala Lumpur, Malaysia; 4 Tropical Infectious Diseases Research and Educational Centre (TIDREC), University of Malaya, Kuala Lumpur, Malaysia; London School of Hygiene and Tropical Medicine, United Kingdom

## Abstract

**Background:**

Demographic, economic and behavioural factors are central features underpinning the successful management and biological control of dengue. This study aimed to examine these factors and their association with the seroprevalence of this disease.

**Methodology:**

We conducted a cross-sectional telephone survey of households in a 3 km radius of the schools where we had conducted serological tests on the student population in a previous study. Households were surveyed about their socio-demographics, knowledge, practices, and Health Belief Model (HBM) constructs. The results were then associated with the prevalence rate of dengue in the community, as marked by IgG seropositivity of the students who attended school there.

**Results:**

A total of 1,400 complete responses were obtained. The community's IgG seropositivity was significantly positively associated with high household monthly income, high-rise residential building type, high surrounding vegetation density, rural locality, high perceived severity and susceptibility, perceived barriers to prevention, knowing that a neighbour has dengue, frequent fogging and a higher level of knowledge about dengue. In the multivariate analyses, three major correlates of the presence of IgG seropositivity in the community: (1) high-rise residential apartment house type or condominium buildings; (2) the main construct of the HBM, perceived severity and susceptibility; and (3) the additional constructs of the HBM, lack of preventive measures from the community level and having a neighbour with dengue as a cue to action. Weak correlations were found between self-practices to prevent dengue and the level of dengue seropositivity in the community, and between HBM constructs and knowledge (r = 0.09).

**Conclusions:**

The residential environment factor and the constructs of the HBM are useful and important elements in developing interventions to prevent and control dengue. The study also sheds light on the importance of the need for approaches that ensure the translation of knowledge into practice.

## Author Summary

Demographic, economic and behavioural factors are important to successful control of dengue. This study aimed to examine these factors and their association with the prevalence rate of dengue in a community marked by IgG seropositivity of students attending schools there, as assessed in our previous study. Telephone interviewing was used to try to reach the households in a 3 km radius of the schools where serological tests were conducted. Results showed three major correlates of the presence of IgG seropositivity in the community: (1) high-rise residential apartment house type or condominium buildings; (2) the main construct of the HBM, perceived severity and susceptibility; and (3) the additional constructs of the HBM, lack of preventive measures at the community level and having a neighbour with dengue as a cue to action. The residential environment factor and the constructs of the HBM are useful and important elements in developing interventions to prevent and control dengue.

## Introduction

The incidence of dengue has grown dramatically in recent decades [1]. Over 2.5 billion people, 40% of the world's population, are at risk from dengue, and each year an estimated 50–100 million dengue infections are reported worldwide [1]. The disease is currently endemic in more than 125 countries in Africa, the Americas, the Eastern Mediterranean, Southeast Asia and the Western Pacific [1]. Since the first reported case of dengue fever in Malaysia in 1902, dengue has remained a serious public health problem in this country. Malaysia has experienced several major outbreaks, which were reported in 1974, 1978, 1982 and 1990 [2]. Likewise in Malaysia, the incidence of dengue fever and its more severe forms have increased dramatically in recent decades. The incidence rate of dengue shows an increasing trend from 44.3 cases/100,000 population in 1999 to 181 cases/100,000 population in 2007 [3]. High dengue IgG seropositivity (>91%) was found in a sample of Malaysian adults aged 35 to 74 years old in a recent study [4]. Serological tests conducted in Lundu District in Sarawak, Malaysia, found that in >23% of 215 samples, individuals had a history of dengue [5]. In another study, a nationwide sample of 1,410 children aged 7 to 18 years was surveyed and 11.0% were found to be positive for dengue IgG (Tiong et al., unpublished data).

Among the most frequently applied methods of controlling or preventing dengue is the control of dengue virus transmission through mosquito reduction activities. Human behaviour is an important contributor to creating breeding grounds for mosquitoes and sustaining mosquito populations [6]. It has long been recognized that socio-demographic characteristics, beliefs and practices about dengue have an impact on dengue prevention and control. The basic concepts of the Health Belief Model (HBM) feature individual consideration of the likelihood (susceptibility) and seriousness (severity) of illness and the capacity of the individual to adopt the desired behaviour to prevent it [7]. Since its original conception, two additional concepts have been added to the HBM: self-efficacy, or one's confidence in the ability to successfully perform an action; and cues to action, external events that prompt readiness to make a health change [8]. A handful of studies have specifically applied the HBM in attempts to understand perceptions of risk and sustained dengue prevention [9]–[11]. In the context of health communication strategies for dengue prevention and control, the HBM provides a framework for understanding how to effectively structure messages and influence behavioural change [12].

Previous studies have primarily focused on investigating the constructs of the HBM in conceptualizing health beliefs and knowledge about the threat of dengue, the barriers to engaging in the desired preventive behaviour, and the subsequent development of self-efficacy to initiate the desired behaviours. This study differs from previous field investigations in that it focuses on investigating the HBM constructs and their association with the seroprevalence of dengue virus-specific IgG in the community. To the best of our knowledge, such a study has never been conducted elsewhere.

## Methods

### Seroprevalence data

The seroprevalence data were obtained from our previous study (Tiong et al., unpublished data). Between 2008 and 2009, anonymized serum samples of school children, aged 7 to 18, from 26 schools throughout Malaysia were examined for dengue virus-specific IgG. Dengue IgG capture ELISA (Standard Diagnostics, Korea, Cat. no. 11EK10) was used to test for the presence of anti-dengue-specific IgG antibodies. Of the 1,411 samples from 26 schools, 156 (11%) were positive for dengue-specific IgG antibodies. The prevalence of seropositivity for dengue-specific IgG in the student population, calculated for each school, ranged from 0% to 25±1%. The percentage of seropositivity for dengue-specific IgG in the surrounding community (within 3 km) was assumed to be the same as that of the student population in the nearby school. Households within a 3 km radius of a school were therefore assigned a corresponding percentage of seropositivity for dengue-specific IgG. In the current study, the level of IgG seropositivity in the study participants was categorized as being ‘absent’ or ‘present’. Further, a K-means cluster analysis procedure was also used to identify groups by the percentage of positive IgG results in the study participants. The households in a 3 km radius of the schools were surveyed about their knowledge and health belief constructs, the results of which were associated with the absence or presence of seropositivity and with the seropositivity groups identified in cluster analysis.

The seroprevalence of dengue amongst the students was used as a surrogate for the prevalence of dengue in the community, as students were not likely to have extensively travelled outside their respective communities. The community around the school was chosen because Malaysian schools usually admit only those students living within a 10 km radius of the respective school. Because of the unmanageably large number of households within a 10 km radius of the schools, this study surveyed only those households within a 3 km radius.

### Sampling frame

The study samples for the present study were households within a 3 km radius of the schools. Residential communities within a 3 km radius of the schools were first identified. Subsequently, we performed a cross-sectional study by contacting all households in the identified residential community with a registered landline telephone (which served as the study sample of the population). To be eligible for a telephone interview, participants had to be Malaysian, aged between 18 and 60 years old, and resident in the contacted household. Only one person per household was surveyed. If more than one eligible person was found in a household, one person was selected randomly by using a random number table. Interviews were conducted between 5.30 p.m. and 10.00 p.m. on weekdays in order to avoid over-representation of unemployed participants, and from 10.00 a.m. to 7.00 p.m. on weekends or on public holidays. Interviewers made three attempts to call unanswered telephones on different days before regarding them as non-responses.

### Survey assessment process

After the survey questions were constructed, a panel of experts, consisting of four members, was assembled to investigate the content validity of the survey. Two parameters were measured in the content validation study: (1) the necessity of each survey item, and (2) the clarity of each survey statement. The panel was asked to comment independently on the necessity and clarity of the items in order to calculate the content validity ratio (CVR) and the content validity index (CVI), respectively. The necessity of the items was assessed by using a three-point rating scale: (1) essential; (2) useful, but not essential; and (3) not necessary. The clarity of the items was also assessed by using a 3-point rating scale: (1) clear, (2) item needs revision, and (3) not clear. Following the experts' assessments, a CVR for the total scale was computed. The CVR in this study for the total scale was 0.61, indicating a satisfactory result. For the necessity parameter, all CVRs were 1.0, thus leading to a CVI of 1.0. The overall CVI for the clarity of survey statements was 0.87. A satisfactory level of agreement was found (CVI>0.80) among panellists, suggesting that the scale had good content validity [13]. Several questions were rephrased, as suggested by the panellists, to improve clarity. The modified questionnaire was executed in the form of a preliminary study, which included a random sample of 50 people to investigate the possible problems of the questionnaire and its reliability.

### Survey questions

The questionnaire inquired about socio-demographic characteristics, house and surrounding environment, beliefs regarding dengue fever, self-reported preventive practices against dengue fever and knowledge of dengue fever.

Belief questions were based on the HBM constructs [7]:

1. *Perceived threat* consists of two parts: perceived susceptibility and perceived severity of dengue fever, where perceived susceptibility assesses one's subjective perception of the risk of contracting dengue fever, and perceived severity assesses feelings concerning the seriousness of dengue fever. Perceived threat was measured on a scale of 1–10, with a higher score indicating greater barriers.
2. *Perceived barrier* examines perceptions of barriers to prevent dengue fever among respondents. This was also measured on a scale of 1–10, with a higher score indicating greater barriers.
3. *Self-efficacy* is the belief in being able to successfully execute dengue prevention behaviour. Self-efficacy was measured by a 4-point Likert scale that ranged from 1 (*strongly agree*) to 4 (*strongly disagree*).
4. *Other contracts and cues to action* include mosquito problems, dengue fever cases reported by neighbours, fogging, events that motivate people to take action, community participation and authority enforcement; these considerations affect an individual's perceptions and thus indirectly influence health-related behaviour (on a 4-point Likert scale ranging from *strongly agree* to *strongly disagree*).

Self-reported practices for the prevention and control of dengue fever, namely (1) prevention of mosquito breeding and (2) prevention of mosquito bites, were assessed by using 6-item and 7-item questions, respectively. The options for practices (not at all, rarely, sometimes, often, and not applicable) were assigned penalty points of 4, 3, 2, 1 and 0, respectively. The possible scores ranged from 0 to 36 for mosquito breeding preventive practices, and 0 to 28 for mosquito bite preventive practices. A higher number of penalty points indicates fewer preventive practices. As the dengue prevention scales were newly developed, an initial pilot test was performed to ensure test-retest reliability, the result of which was found to be acceptable (correlation coefficient >0.70). Cronbach's alpha measurements were also performed. Cronbach's alpha coefficients for prevention of mosquito breeding and mosquito bites were 0.791 and 0.898, respectively, demonstrating good internal consistency.

The scale for the measurement of knowledge of dengue fever consisted of 43 items. For each statement, the respondents could choose between three response categories: yes, no and don't know. For the analyses, the responses were scored as 1 for a correct response and 0 for an incorrect response or a ‘don't know’ response. Possible scores ranged from 0 to 43. The higher scores indicate greater knowledge about dengue fever. Cronbach's alpha was 0.916, showing high internal consistency.

### Ethical considerations

The study was approved by the Medical Ethics Committee of the University of Malaya Medical Centre, Kuala Lumpur, Malaysia (MEC Ref No. 896.15). Due care was taken to ensure that all those who agreed to participate in the study did so voluntarily. Respondents were assured that their responses would remain confidential and anonymous, and that they were free to withdraw from the interview at any time. As written informed consent is not practical in a telephone survey, verbal informed consent was obtained from the respondents before the beginning of an interview. The verbal consent procedure was approved by the Medical Ethics Committee.

### Statistical analyses

The 26 schools throughout Malaysia were sampled randomly from six zones (North, East, West, Centre and South of Peninsular Malaysia, as well as Sabah in East Malaysia). Therefore, conventional statistical analyses with underlying distributional assumptions were inappropriate for variance estimation and statistical testing because of the multistage probability sampling design. Sampling weights were incorporated into the analyses to produce representative estimates. Each observation corresponding to the IgG of the respective school was proportionally weighted according to the overall school samples in the respective zone before analysis to account for the complex sampling design. The dependent variable (percentage of positive IgG) corresponding to each respondent was compared with the independent variable (socio-demographic characteristics, HBM constructs, dengue prevention practices and knowledge) by using t-test analysis, analysis of variance or chi-square analysis to see how the variables were associated independently of level of seroprevalence. In multivariate analysis, multiple linear regression standard errors are computed by using a sandwich estimator. A generalized linear model was used in which the outcome was the presence of a level of dengue seroprevalence *vs*. its absence. The covariance matrix was estimated by using the robust estimator method. This sandwich standard error estimator assumes independence of the clusters. Correlations of individual level data were conducted with Spearman's rank correlation coefficient to examine the association between self-reported practices to prevent mosquito breeding and mosquito bites and (1) HBM constructs and (2) knowledge score.

Effect sizes of 0.20 or lower were considered to demonstrate ‘small’ relationships, while those ranging from 0.30 to 0.50 were considered to demonstrate ‘medium’ relationships and those of 0.80 or greater ‘large’ relationships [14]. All statistical analyses were performed with SPSS 16.0 (SPSS Inc., Chicago, IL). In all analyses, a *P*-value of less than 0.05 was considered statistically significant.

## Results

**Figure 1:**
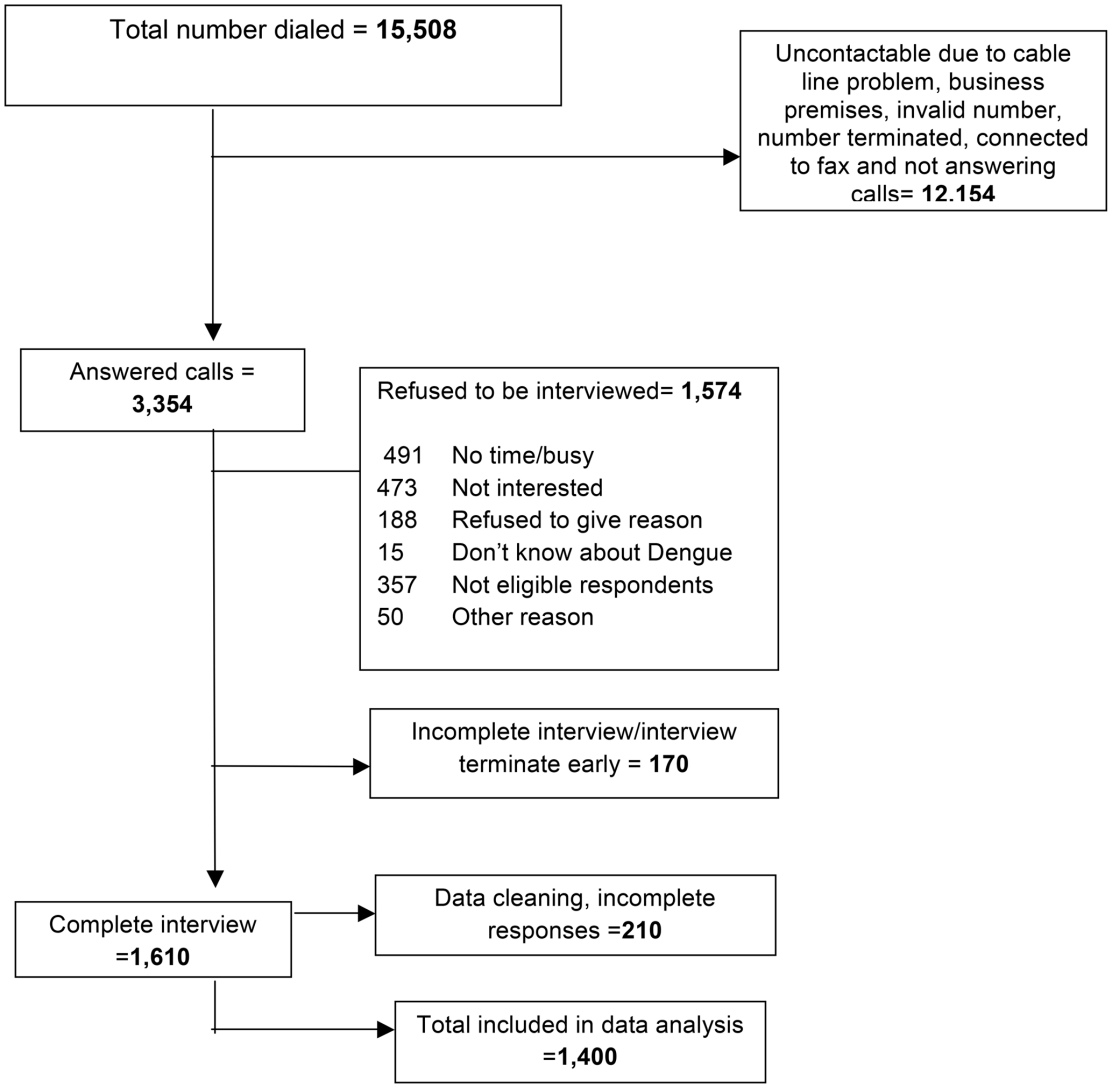
Illustration of the computer-assisted telephone interviewing survey process.

A total of 15,508 registered home telephones were identified for households within a 3 km radius of the schools where the student population was tested for dengue IgG seropositivity. Attempts were made to call the numbers between 19 March 2011 and 20 May 2012. Of a total of 5,027 households that were successfully contacted, 1,610 responded to the survey. It was not possible to contact all households for various reasons, including calls not being answered, calls answered by an answering machine, the telephone number being engaged, the number being connected to a fax machine, and service termination. The most common reasons for refusal to participate were 'too busy' and 'not interested'. The data were edited to find and remove incomplete responses, and a final total of 1,400 complete responses were obtained and analysed. The response rate was 27.8% (computed as the number of completed responses divided by the number of eligible and contacted households; Figure 1).

### Demographic, housing and surrounding environment

There were no significant differences in the percentage of positive IgG results in the community by ethnicity, gender, educational level or household size (Table 1). Dengue-specific IgG was present in 83.0% of the group earning a household income of more than MYR4000 monthly (USD1313) and in 70.4% of the group earning a household income of less than MYR2000 monthly (USD657; *P*<0.001). A significantly higher proportion of respondents from the community presenting dengue IgG seropositivity were found to be residing in high-rise residential apartment or condominium buildings than in single (67.9%) or terraced (61.1%) houses.

**Table 1:** Multivariate logistic regression analysis of variables associated with being seropositive for IgG (N = 1400).

Characteristic	Presence of dengue IgG (N = 923) n (%)	Absence of dengue IgG (N = 477) n (%)	Univariate OR	Multivariate ordinal logistic regression
			Presence *vs*. absence of dengue seroprevalence [Unadjusted OR (95% CI)]	Presence *vs*. absence of dengue seroprevalence [Adjusted OR (95% CI)]
***Personal background***				
Age, years				
Below 20	79(64.8)	43(35.2)	0.95(0.64–1.42)	
21 to 40	281(66.4)	142(33.6)	1.03(0.80–1.31)	-
41 and above	563(65.8)	292(34.2)	Reference	
Gender				
Male	292(65.6)	153(34.4)	1.02(0.81–1.30)	-
Female	631(66.1)	324(33.9)	Reference	
Ethnicity¶				
Malay	598(67.2)	292(32.8)	1.24(0.92–1.67)	
Chinese	175(64.6)	96(35.4)	1.10(0.77–1.59)	-
Indian	144(62.3)	87(37.7)	Reference	
Average household monthly income†				
<MYR2000	420(70.4)	177(29.6)	0.50(0.28–0.84)	0.49(0.23–0.99)*
MYR2001-4000	420(59.7)	283(40.3)	0.30(0.18–0.52)	0.34(0.22–0.86)*
>MYR4000	83(83.0)	17(17.0)	Reference	Reference
Educational level				
No formal education	50(66.7)	25(33.3)	0.92(0.54–1.56)	
Primary	170(66.4)	86(33.6)	0.91(0.64–1.28)	-
Secondary	472(64.5)	260(35.5)	0.83(0.63–1.09)	
Tertiary	231(68.5)	106(31.5)	Reference	
***Household size***				
Mean (SD) number of households	4.8(2.4)	4.6 (2.4)	1.06 (0.98–1.14)	-
***Self-reported house and surrounding environment***				
House type				
High rise	80(85.1)	14(14.9)	2.70(1.49–4.87)	2.67(1.10–5.65)**
Terrace house	396(61.1)	252(38.9)	0.74(0.59–0.93)	0.98(0.65–1.54)
Single/village house	447(67.9)	211(32.1)	Reference	Reference
Density of vegetation/plants				
None	55(82.1)	12(17.9)	1.36(0.69–2.67)	1.61(0.80–4.25)
Low	413(62.0)	253(38.0)	0.49(0.36–0.63)	0.59(0.44–1.67)
Moderate	189(58.7)	133(41.3)	0.42(0.30–0.59)	0.62(0.40–0.98)*
A lot	266(77.1)	79(22.9)	Reference	Reference
Locality				
Urban	373(55.4)	300(44.6)	0.38(0.28–0.52)	0.40(0.30–0.74)**
Suburban	332(75.1)	110(24.9)	0.93(0.66–1.31)	0.68(0.35–1.29)
Rural	218(76.5)	67(23.5)	Reference	Reference
***Health beliefs***				
**Perceived severity (scale 1–10)**				
Mean (SD) severity score	7.45(2.90)	6.88(3.58)		
Severity of dengue				
1–2	110(49.3)	113(50.7)	0.57(0.42–0.77)	0.60(0.45–1.48)
3–4	39(69.6)	17(30.4)	1.34(0.74–1.42)	1.69(0.68–4.98)
5–6	100(76.3)	31(23.7)	1.88(1.23–2.90)	1.62(0.98–2.74)
7–8	215(81.7)	48(18.3)	2.62(1.85–3.70)	1.84(1.25–2.87)***
9–10	459(63.1)	268(36.9)	Reference	Reference
**Perceived susceptibility (scale 1–10)**				
Mean (SD) susceptibility score	5.40(2.32)	5.18(2.52)		
Susceptibility of dengue				
1–2	140(57.9)	102(42.1)	0.59(0.36–0.95)	1.26(0.82–2.59)
3–4	80(82.5)	17(17.5)	2.01(1.03–3.91)	4.50(1.95–10.99)**
5–6	414(64.3)	230(35.7)	0.77(0.49–1.20)	1.30(0.89–2.10)
7–8	214(69.0)	96(31.0)	0.95(0.59–1.54)	1.61(0.92–2.85)
9–10	75(70.1)	32(29.9)	Reference	Reference
**Perceived barriers to sustained prevention (scale 1–10)**				
Mean (SD) barrier score	4.47(2.53)	3.79(2.70)		
Barriers to sustained dengue prevention				
1–2	253(54.1)	215(45.9)	0.40(0.23–1.70)	0.50(0.24–1.25)
3–4	152(80.0)	38(20.0)	1.36(0.72–2.58)	1.30(0.56–2.95)
5–6	326(67.8)	155(32.2)	0.71(0.41–1.26)	0.98(0.45–1.95)
7–8	139(73.2)	51(26.8)	0.93(0.50–1.73)	0.85(0.35–1.29)
9–10	53(74.6)	18(25.4)	Reference	Reference
**Self-efficacy**				
Lack of self-efficacy in taking preventive measures				
Strongly agree/agree	448(78.3)	124(21.7)	2.69(2.11–3.42)	1.48(0.65–2.38)
Strongly disagree/disagree	475(57.4)	353(42.6)	Reference	Reference
**Cues to action**				
Mosquito problem in neighbourhood				
None	267(62.5)	160(37.5)	0.30(0.18–0.50)	1.80(0.58–3.95)
Low	417(66.1)	214(33.9)	0.35(0.21–0.58)	0.93(0.45–1.97)
Moderate	132(61.1)	84(38.9)	0.28(0.16–0.49)	0.45(0.20–0.97)*
Severe	107(84.9)	19(15.1)	Reference	Reference
Neighbour with dengue fever				
Yes	204(79.4)	53(20.6)	2.27(1.64–3.14)	1.45(1.28–2.53)*
No	719(62.9)	424(37.1)	Reference	Reference
Frequency of fogging in neighbourhood				
None	204(54.3)	172(45.7)	1.27(0.07–0.24)	0.09(0.07–0.50)***
Rarely	458(65.5)	241(34.5)	0.20(0.11–0.38)	0.27(0.20–0.65)**
Occasionally	149(74.1)	52(25.9)	0.31(0.16–0.60)	0.48(0.25–0.96)*
Often	112(90.3)	12(9.7)	Reference	Reference
Lack of preventive measures at community level				
Strongly agree/agree	468(75.6)	151(24.4)	2.22(1.76–2.80)	3.1(1.58–3.85)***
Strongly disagree/disagree	455(58.3)	326(41.7)	Reference	Reference
Lack of preventive measures from authorities				
Strongly agree/agree	303(74.1)	106(25.9)	1.71(1.32–2.21)	1.02(0.58–1.98)
Strongly disagree/disagree	620(62.6)	371(37.4)	Reference	Reference
***Practices***				
Self-practices in prevention of mosquito breeding (0-36)				
Mean penalty points (SD) ‡	13.8(6.7)	13.2(5.9)		
0–12	441(66.4)	223(33.6)	0.70(0.36–0.99)	0.88(0.54–2.01)
13–25	445(64.9)	241(35.1)	0.65(0.34–1.24)	0.45(0.35–1.59)
26–36	37(74.0)	13(26.0)	Reference	Reference
Self-practices in prevention of mosquito bite (0–28)				
Mean penalty points (SD) ‡	20.4(7.8)	19.2(8.4)		
0–10	137(58.5)	97(41.5)	0.71(0.53–0.95)	1.89(0.65–2.38)
11–20	135(71.4)	54(28.6)	1.25(0.89–1.75)	0.54(0.49–0.98)*
21–28	651(66.6)	326(33.4)	Reference	Reference
***Knowledge (0–43)***				
Mean knowledge score (SD) §	28.1(7.0)	26.5(8.8)		
0–14	43(43.4)	56(56.6)	0.35(0.23–0.55)	0.30(0.25–0.65)***
15–30	504(67.0)	248(33.0)	0.94(0.74–1.18)	0.98(0.65–1.39)
21–42	376(68.5)	173(31.5)	Reference	Reference

¶ Other ethnic groups not included in analysis; number does not total 1400.

† 1 US Dollar  = 3.0 Malaysian Ringgit (MYR).

‡ A higher score indicates decreasing preventive practices.

§ A higher score indicates increasing knowledge.

***Association is significant at the 0.001 level.

**Association is significant at the 0.01 level.

*Association is significant at the 0.05 level.

There were no clear ascending or descending trends between the proportion of respondents with dengue IgG present and the surrounding vegetation density levels reported, but participants reporting no vegetation in the surrounding environment were found to have the highest proportion of dengue IgG present (82.1%) compared with those reporting a high vegetation density in the surrounding environment (77.1%). The proportion of respondents with dengue IgG present was significantly higher among those from high-rise apartment or condominium buildings who reported moderate surrounding vegetation density (66.0%) than it was for those who reported no vegetation density (15.0%).

The proportion of respondents with dengue IgG present was also significantly higher in rural than in urban areas (76.5% versus 55.4%). A higher proportion of rural respondents (44.7%) reported a high level of vegetation density in their surrounding environment than did their urban counterparts (15.8%).

### Health beliefs

The mean (SD) rating of perceived severity of dengue fever on a scale from 1 to 10 was 7.45 (SD = 2.90) for respondents from communities where positive dengue IgG results were present and 6.88 (3.58) for respondents from communities where positive dengue IgG results were absent. Among respondents from communities where dengue IgG was present, the majority (81.7%) rated the severity of dengue fever as 7 and 8. The mean (and SD) rating of the perceived susceptibility score was 5.40 (SD = 2.32) for respondents from communities where dengue IgG was present and 5.18 (SD = 2.52) for respondents from communities where dengue IgG was absent. The mean (SD) rating for the perceived barriers to sustain dengue prevention was also higher for respondents from communities where dengue IgG was present (4.48, SD = 2.39) than for respondents from communities where positive dengue IgG was absent (3.79, SD = 2.70). A higher proportion of respondents from communities where positive dengue IgG results were present reported that they either strongly agreed or agreed (78.3%) that they lacked efficacy in taking preventive measures, as compared with respondents from communities where positive dengue IgG results were absent (21.7%).

Mosquito problems in the neighbourhood were reported as severe by 84.9% respondents from communities where dengue IgG was present compared with only 15.1% among respondents from communities where dengue IgG was absent. A higher proportion of respondents from communities where dengue IgG seropositivity was present (79.4%) reported that they were aware that dengue was in their community, as compared with respondents from communities where dengue IgG was absent (20.6%). Respondents from communities where dengue IgG was present reported a higher frequency of mosquito fogging in their community than did those from communities where dengue IgG was absent.

A higher proportion of respondents from communities where positive dengue IgG results were present reported that they either strongly agreed or agreed (75.6%) that there was a lack of preventive measures at the community level, as compared with respondents from communities where positive IgG results were absent (24.4%). Similarly, a higher proportion of respondents from communities where IgG was present reported that they either strongly agreed or agreed (74.1%) that there was a lack of preventive measures from the authorities, as compared with respondents from communities where IgG was absent (25.9%).

### Practices and knowledge

Thirty-seven per cent of respondents (350 of 944) did not periodically examine mosquito breeding places in their surroundings, 20% (167 of 849) did not cover their water storage containers, and 9% (86 out of 959) did not periodically change water stored at home. The total responses do not add up to 1,400 because some respondents did not practise storing water at home. A higher proportion of urban respondents (53.9%) did not practise storing water compared with their rural counterparts (34.4%). The mean (SD) penalty points for self-reported practices to prevent mosquito breeding was slightly higher among respondents from communities where positive dengue IgG results were present (13.8, SD = 6.7) than among respondents from communities where positive IgG results were absent (13.2, SD = 5.9).

Using cluster analysis, we identified three distinct percentages of positive IgG seropositivity clusters. Table 2 shows the characteristic differences of respondents in three subgroups by percentage level of IgG (%) identified by cluster analysis. Cluster I, consisting of nine schools, has the lowest mean percentage of IgG (2.37, SD±2.39), whereas cluster III (total of six schools) has the highest mean percentage of IgG (25.2, SD±3.02). Cluster III had a higher proportion of high-rise house types (15.6%) and village houses (61.7%). Across clusters (Cluster I to Cluster III), an increasing proportion of respondents reported “a lot” of vegetation density. Likewise, an increasing proportion of respondents across clusters agreed that there was a lack of preventive measures at the community level and a lack of self-practice to prevent mosquito bites, as well as fogging. In contrast, we found a decreasing proportion of respondents with a high knowledge score from Cluster I to Cluster III.

**Table 2:** Characteristic differences of respondents in three subgroups by percentage levels of IgG (%) identified by cluster analysis (N = 1400).

Characteristic	Cluster I N = 858 n (%)	Cluster II N = 362	Cluster III N = 180	ANOVA for continuous and χ^2^ for categorical variables *P*-value
Number of schools	9	11	6	
Mean (SD) percentage of IgG	2.37(2.39)	9.51(2.59)	25.2(3.02)	<0.001
Percentage range of IgG	0 to 5.9	6.5 to 14.3	21.7 to 33.9	
***Personal background***				
Age, years				
Mean age (SD) φ	45.1(15.1)	44.4(16.0)	44.4(18.1)	ns
Gender				
Male	280(32.6)	110(30.4)	55(30.6)	
Female	578(67.4)	252(69.6)	125(69.4)	ns
Ethnicity¶				
Malay	523(61.3)	209(58.1)	158(88.3)	
Chinese	151(17.7)	107(29.7)	13(7.3)	<0.001
Indian	179(21.0)	44(12.2)	8(4.5)	
Average household monthly income†				
<MYR2000	363(42.3)	143(39.5)	91(50.6)	
MYR2001-4000	433(50.5)	198(54.7)	72(40.0)	0.027
>MYR4000	62(7.2)	21(5.8)	17(9.4)	
Educational level				
No formal education	51(5.9)	19(5.2)	5(2.8)	
Primary	153(17.8)	75(20.7)	28(15.6)	
Secondary	438(51.0)	193(53.3)	101(56.1)	ns
Tertiary	216(25.2)	75(20.7)	46(25.6)	
***Household size***				
Mean (SD) number of householdsφ	3.18(2.0)	3.21(1.95)	3.02(2.08)	ns
***Self-reported house and surrounding environment***				
House type				
High rise	52(6.1)	14(3.9)	28(15.6)	<0.001
Terrace house	425(49.5)	182(50.3)	41(22.8)	
Single/village house	381(44.4)	166(45.9)	111(61.7)	
Density of vegetation/plants				
None	40(4.7)	18(5.0)	9(5.0)	
Low	413(48.1)	186(51.4)	67(37.2)	
Moderate	229(26.7)	54(14.9)	39(21.7)	<0.001
A lot	176(20.5)	104(28.7)	65(36.1)	
Locality				
Urban	352(41.0)	247(68.2)	74(41.1)	
Suburban	318(37.1)	51(14.1)	73(40.6)	<0.001
Rural	188(21.9)	64(17.7)	33(18.3)	
***Health beliefs***				
**Perceived severity (scale 1–10)**				
Mean (SD) severity scoreφ	7.1(3.3)	7.6(2.9)	7.3(3.0)	0.037
Severity of dengue				
1–2	158(18.4)	41(11.3)	24(13.3)	
3–4	31(3.6)	13(3.6)	12(6.7)	
5–6	69(8.0)	42(11.6)	20(11.1)	<0.001
7–8	191(22.3)	34(9.4)	38(21.1)	
9–10	409(47.7)	232(64.1)	86(47.8)	
**Perceived susceptibility (scale 1–10)**				
Mean (SD) susceptibility scoreφ	5.3(2.5)	5.5(2.2)	5.2(2.3)	ns
Susceptibility of dengue				
1–2	162(18.9)	49(13.5)	31(17.2)	
3–4	63(7.3)	21(5.8)	13(7.2)	
5–6	363(42.3)	193(53.3)	88(48.9)	0.037
7–8	207(24.1)	69(19.1)	34(18.9)	
9–10	63(7.3)	30(8.3)	14(7.8)	
**Perceived barriers to sustained prevention (scale 1–10)**				
Mean (SD) barrier scoreφ	4.2(2.6)	4.6(2.5)	3.7(2.6)	<0.001
Barriers to sustained dengue prevention				
1–2	292(34.0)	95(26.2)	81(45.0)	
3–4	119(13.9)	39(10.8)	32(17.8)	
5–6	281(32.8)	164(45.3)	36(20.0)	<0.001
7–8	125(14.6)	44(12.2)	21(11.7)	
9–10	41(4.8)	20(5.5)	10(5.6)	
**Self-efficacy**				
Lack of self-efficacy in taking preventive measures				
Strongly agree/agree	339(39.5)	140(38.7)	93(51.7)	
Strongly disagree/disagree	519(60.5)	222(61.3)	87(48.3)	0.007
**Cues to action**				
Mosquito problem in neighbourhood				
None	293(34.1)	73(20.2)	61(33.9)	
Low	368(42.9)	203(56.1)	60(33.3)	
Moderate	138(16.1)	51(14.1)	27(15.0)	<0.001
Severe	59(6.9)	35(9.7)	32(17.8)	
Neighbour with dengue fever				
Yes	130(15.2)	64(17.7)	63(35.0)	
No	728(84.8)	298(82.3)	117(65.0)	<0.001
Frequency of fogging in neighbourhood				
None	276(32.2)	57(15.7)	43(23.9)	
Rarely	406(47.3)	219(60.5)	74(41.4)	
Occasionally	125(14.6)	52(14.4)	24(13.3)	<0.001
Often	51(5.9)	34(9.4)	39(21.7)	
Lack of preventive measures at community level				
Strongly agree/agree	362(42.2)	161(44.5)	96(53.3)	
Strongly disagree/disagree	496(57.8)	201(55.5)	84(46.7)	0.023
Lack of preventive measures from authorities				
Strongly agree/agree	245(28.6)	117(32.3)	47(26.1)	ns
Strongly disagree/disagree	613(71.4)	245(67.7)	133(73.9)	
***Practices***				
Self-practices in prevention of mosquito breeding (0–36)				
Mean penalty points (SD) ‡ ^φ^	13.7(6.6)	13.5(5.9)	13.4(6.5)	ns
0–12	409(47.7)	164(45.3)	91(50.6)	
13–25	420(49.0)	186(51.4)	80(44.4)	ns
26–36	29(3.4)	12(3.3)	9(5.0)	
Self-practices in prevention of mosquito bite (0–28)				
Mean penalty points (SD) ‡ ^φ^	21.3(6.9)	16.6(9.8)	20.9(6.9)	<0.001
0–10	92(10.7)	124(34.3)	18(10.0)	
11–20	110(12.8)	39(10.8)	40(22.2)	
21–28	656(76.5)	199(50.0)	122(67.8)	<0.001
***Knowledge (0–43)***				
Mean knowledge score (SD) § ^φ^	27.6(7.6)	26.5(8.4)	29.2(6.7)	<0.001
0–14	50(5.8)	44(12.2)	5(2.8)	
15–30	477(55.6)	188(51.9)	87(48.3)	<0.001
21–42	331(38.6)	130(35.9)	88(48.9)	

¶ Other ethnic groups not included in analysis; number does not total 1400.

† 1 US Dollar  = 3.0 Malaysian Ringgit (MYR).

‡ A higher score indicates decreasing preventive practices.

§ A higher score indicates increasing knowledge.

***Association is significant at the 0.001 level.

**Association is significant at the 0.01 level.

*Association is significant at the 0.05 level.

φ One-way analysis of variance (ANOVA).

The Spearman correlation between scores for self-prevention practices and the constructs of the HBM, and between scores for self-preservation practices and knowledge scores, are shown in Table 3. The effect size, Spearman's rho, indicates that the correlations between self-prevention practices (both prevention against mosquito breeding and against mosquito bites) and the constructs of the HBM can be considered a small effect, according to Cohen's criteria on effect size. Significant small effect sizes were also found in the correlations between knowledge scores and preventive practices.

**Table 3:** Correlation analyses between self-reported practices to prevent mosquito breeding and mosquito bites and (1) HBM constructs, and (2) knowledge score (N = 1400).

	Spearman's rho	
	Prevention of mosquito breeding score‡	Prevention of mosquito bite score‡
Health beliefs		
Severity score	0.076**	−0.104**
Susceptibility score	−0.040	−0.089**
Barriers to prevention score	−0.004	−0.294**
Lack of self-efficacy	0.168**	0.141**
Mosquito problem	0.133**	−0.048
Dengue in the community	−0.075**	−0.033
Fogging frequency	0.065*	−0.045
Lack of preventive measures in community	−0.143**	0.169**
Lack of preventive measures from authorities	−0.045	0.128**
Knowledge score	−0.061*	0.023*

‡ A higher score indicates decreasing preventive practices.

**Correlation is significant at the 0.01 level (2-tailed).

*Correlation is significant at the 0.05 level (2-tailed).

### Multivariate logistic regression findings

Findings from multivariate logistic regression analysis (Table 1) indicate that the income group of MYR2001–4000 monthly (USD757-1313) was less likely than the income group above MYR4000 monthly (odds ratio [OR] = 0.34, 95% CI, 0.22–0.86, *P*<0.01) to be seropositive for dengue IgG. Likewise, the income group below MYR2000 monthly was also less likely than the group above MYR4000 monthly (USD1313) to be seropositive for dengue IgG (OR = 0.49, 95% CI, 0.34–0.99, *P*<0.05). Compared with those who reported a high level of vegetation density, those who reported moderate vegetation density (OR = 0.62, 95% CI, 0.40–0.98, *P*<0.05) had a lower likelihood of being seropositive for dengue IgG. Those living in an urban area (OR = 0.40, 95% CI, 0.30–0.74, *P*<0.001) had a lower likelihood of being seropositive for dengue IgG compared with those living in the reference rural area.

The results of multivariate logistic regression analysis also indicated that the two main constructs of the HBM (perceived severity and susceptibility) were significant correlates of seropositivity for dengue IgG. Those with a lower perceived severity (level of severity 7–8) had a higher likelihood (OR = 1.84, 95% CI, 1.25–2.87, *P*<0.001) of being seropositive for dengue IgG compared with those with a reference level of severity of 9–10. Likewise, those with a lower perceived susceptibility (level 3–4) had a higher likelihood (OR = 4.50, 95% CI, 1.95–10.99, *P*<0.01) of being seropositive for dengue IgG compared with those with a reference level of 9–10.

Having perceived barriers to sustain prevention (on a scale of 1–10) was not found to be significantly associated with being seropositive for dengue IgG in the logistic regression model. Respondents who rated the mosquito problem as 'moderate' had a lower likelihood of being dengue seropositive (OR = 0.45, 95% CI, 0.20–0.97, *P*<0.05) than did those who rated it as 'severe'. Respondents who knew dengue was in their community had an increased likelihood of being dengue seropositive (OR = 1.45, 95% CI, 1.28–2.53, *P*<0.01). There was a significant relationship between the gradual increase in the likelihood of being seropositive for dengue IgG and the frequency of fogging. Self-reported practices to prevent mosquito breeding was not a significant correlate of being seropositive for dengue IgG; however, having lower mean penalty points in the prevention of mosquito bites was associated with a lower likelihood of being seropositive for dengue IgG (OR = 0.54, 95% CI, 0.49–0.98, *P*<0.001). Lack of preventive measures from authorities was not a significant correlate of being seropositive for dengue IgG; however, lack of preventive measures at the community level was significantly associated with IgG seropositivity (OR = 3.1 95% CI, 1.58–3.85, *P*<0.001). A low mean knowledge score (0–14) was associated with a lower likelihood of seropositivity for dengue IgG (OR = 0.30, 95% CI, 0.25–0.65, *P*<0.001).

## Discussion

In this study, income levels were significantly associated with seropositivity for dengue IgG in the community. This finding is in contrast to results from a study in Brazil where the prevalence of seropositivity was equally high from the highest to the lowest socio-economic group and thus there were no differences in socio-economic outcomes and dengue seropositivity [15]. However, findings from Singapore [16] have shown that a high incidence of dengue fever was associated with socio-economic and demographic characteristics of the population, such as having a low income, living alone, being female and having no family nucleus. Failure to check the breeding of mosquito larvae was most commonly reported by our study respondents, as other studies have likewise reported [16]–[17]. This finding highlights the importance of educational campaigns to encourage community participation in detecting and eliminating mosquito breeding grounds.

The finding that having a higher income and residing in a high-rise residential apartment or condominium building were significantly associated with dengue IgG seropositivity in this study could be explained by population density effects, as high-rise apartment or condominium buildings are typically accompanied by high population densities where people with a higher socio-economic level reside. High population density is one of the factors recognized for favouring transmission of dengue virus [18]–[19]; hence, the findings suggest that communities in high-rise buildings are at higher risk for contracting a dengue infection. The positive association between the reported vegetation density in the surrounding environment and IgG seropositivity found in this study could be attributed to the increase in sites that favour the breeding of mosquitoes, thus leading to an increase in the number of dengue cases [20]–[22]. In this study, despite the low vegetation density surrounding high-rise buildings, the higher level of community dengue IgG seropositivity in the residents could be due to the popularity of indoor ornamental plants. It has been reported that stagnant water in the pots of indoor ornamental plants are common breeding grounds for *Aedes sp*. mosquitoes in high-rise buildings in Malaysia [23]. Further, another study in Malaysia has shown that *Aedes sp*. mosquitoes live longer in indoor than in outdoor environments [24].

Despite the fact that urban areas contribute substantially to the high incidence of dengue because of their high population densities, a previous study has found that rural areas contribute at least as much to dengue epidemics as cities do [25]. It was reasoned that a poor tap water supply stimulates people to store water in their households, which increases the risk of dengue breeding. Similarly, we found a higher likelihood of community IgG seropositivity in respondents from rural areas. Although water scarcity is rarely a major problem for homes in rural areas or outside major population centres in Malaysia, two-thirds of our rural respondents practised storing water. Therefore, active community engagement and participation is warranted in order to contain the breeding of mosquitoes in water-catching or storage containers among communities where storing water is still a widespread practice [26].

Media coverage and the extent of reporting of events have been found to greatly influence public perception of the severity of the disease [27]. Further, communities that have been exposed to dengue are more aware of its severity and have higher levels of perceived susceptibility [17]. In Malaysia, cases of dengue are often made known to the public and have always received significant media attention. As evident in this study, residents of localities with high IgG seropositivity had a higher likelihood of reporting that they know neighbours who have dengue. Taken together, these factors could have resulted in a stronger perception of dengue as a serious problem among communities where dengue is prevalent, resulting in a higher perceived severity and susceptibility score among respondents where seropositivity is present than among those where it is absent. The overall mean score for perceived severity, at around the midpoint, 5, in a maximum score of 10, indicates the need to modulate public perception about the severity of dengue fever in Malaysia.

The finding of a positive association between perceived barriers to sustaining dengue prevention and the level of IgG seropositivity is in accordance with the HBM, where the perceived barrier to prevention may serve as a demotivator for carrying out preventive measures and may deter behavioural intention to practice, thus leading to an increased incidence of dengue. The results also showed that communities with IgG seropositivity present reported a greater lack of self-efficacy in taking preventive measures against dengue; lack of preventive measures at the community and authority level was similarly reported. Given this finding, efforts should be made in future qualitative studies to gain in-depth understanding and to identify the underlying reasons for low self-efficacy, as well as the community and authority barriers to dengue prevention. The practice of insecticide fogging as a preventive measure against dengue is still controversial [28]. While this study was unable to determine a direct association between insecticide fogging and the incidence of dengue, it was observed that the higher the IgG seropositivity of the community, the higher the level of reported mosquito problems and fogging frequency.

Constantianus et al. reported that better preventive practice may not necessarily lead to a reduction in dengue risk, because the specific targets for the reduction of mosquito populations that will result in the desired public health outcomes have not yet been fully defined and are expected to be difficult to achieve [29]. However, in the current study, we found a positive statistically significant association between IgG seropositivity and self-practices to prevent mosquito breeding and mosquito bites in the univariate analyses, despite the small effect, although this was not significant in the multivariate analysis. The significant positive association in this study is perhaps explained by the widely publicized incidence of dengue cases in areas of high prevalence, thus provoking a high level of community engagement in dengue preventive measures. This finding may imply that mass media coverage of dengue cases is important in changing public preventive practices against dengue. The association between media frequency and perceived disease severity, which has an impact on health behaviour, was reported by Young et al. [27]. Further, a weak positive correlation coefficient between self-preventive practices and HBM belief constructs may rule out the benefits of health communication messages designed through HBM constructs to communicate awareness about dengue and its control, thus bringing about behaviour change. The findings suggest that action against dengue outbreaks should be integrated with other dengue prevention and control strategies complementary to HBM theory, which is similarly noted in another study [9].

The positive, but weak, association demonstrated between knowledge and self-practices to prevent dengue observed in our study is consistent with results from local [30]–[31] and other studies carried out in numerous countries worldwide [29] [32]–[34]. The weak magnitude of the effect sizes shows that knowledge does not necessarily correspond to practical preventive measures against dengue. An explanation for the positive association could be that people become more knowledgeable when living in communities with a high prevalence of dengue [29]. The results obtained imply that an effective and sustainable strategy for community mobilization to put knowledge into practice may have a direct and sizeable impact. Various strategies and policies directed at effective dengue prevention and control through community mobilization have been suggested. Among these are active community participation in support of school-based health education, monitoring and community empowerment, and the imposition of penalties for mosquito-related offences [35]–[36].

The most prominent finding of the multivariate analysis is that living in high-rise residential apartment or condominium buildings was found to have the highest odds of positive dengue IgG. Prevention and control efforts therefore need to be directed to areas with high-rise residential buildings and a high population density. These results parallel those of several earlier studies [18]–[19]. The second major finding to emerge from the multivariate analysis is that the perceived severity and susceptibility constructs of the HBM are associated with the second-highest odds of perceived likelihood regarding positive dengue IgG. The earlier findings of weak correlational associations between perceived severity and susceptibility and self-prevention practices may imply that these two health belief constructs are mediated through factors other than self-prevention practices in predicting IgG seropositivity. In light of this finding, the mediating factors that moderate the effects of health beliefs and IgG seropositivity merit further examination in future studies. The additional constructs of the HBM, perceived self-efficacy and knowing a neighbour has dengue as cues to action, showed high odds ratios for the likelihood of being IgG seropositive and must be considered in the context of prevention and control strategies. Lack of preventive measures at the community level was associated with the likelihood of being IgG seropositive. Lastly, there was also a significant association with the knowledge score, but with a relatively low odds ratio, which again provides strong support for the lack of translation of knowledge about dengue and preventive practices to real behaviour and preventive practices [37].

The implications of the findings of this study should be interpreted with caution. First, because seropositivity for dengue-specific IgG was not tested in individual participants, we cannot conclude that an individual respondent's level of IgG seropositivity was similar to that in the community near the student population of a school. However, the parallel increase in fogging frequencies in this study, along with the increase in the proportion of the community with IgG seropositivity, may imply that the seroprevalence of dengue amongst the students that was used as a surrogate for the prevalence of dengue in the community accurately portrays the community dengue situation. In Malaysia, fogging is most often performed in dengue-affected areas. Second, the study was conducted only in the communities of the 26 schools where virus-specific IgG was examined; therefore, care must be taken not to generalize beyond the study population. Third, a 3 km radius may not be representative of all of the community children who attend the school; further, dengue may be contracted elsewhere in the region, although the use of student serological testing as a surrogate indictor of community dengue prevalence was meant to minimize this potential because students are not likely to have travelled far from their home and school surroundings. Fourth, households without a landline telephone were not represented in the study; moreover, there are a growing number of households with mobile phones and no landline phones. The fifth limitation of this study is the low response rate of 27.8%, although this is nonetheless common in telephone surveys. The sixth limitation is that although the results may imply an association of the HBM with dengue preventive behaviours, the appropriateness of attitudes conceptualized by the HBM has raised concerns in the case of dengue, where repetitive preventive measures are to be performed on a daily basis and, in particular, the outbreak of dengue is seasonal [38]. Lastly, there is the possibility of bias entailed in general telephone-based surveying, where the data collected were self-reported and may be subject to reporting bias.

### Conclusion

Our multivariate analyses revealed three major correlates of IgG seropositivity that should be the prime focus in dengue prevention and control: (1) high-rise residential apartment or condominium building house type; (2) the main construct of the HBM, perceived severity and susceptibility; and (3) the additional constructs of the HBM, lack of preventive measures at the community level and knowing a neighbour has dengue as a cue to action. These findings also suggest that constructs of the HBM can be integrated as ways of motivating the adoption of preventive practices against dengue, and they may work best by complementing other advocacy and mobilization approaches. Another significant outcome of this study is that it sheds light on the importance of the need for approaches that ensure the translation of knowledge into practice.

Most important, the findings have profound implications for future studies that should seek more accurate and confirmatory evidence by investigating the socio-demographic and HBM constructs and their association with individual levels of IgG seropositivity.

## Supporting Information

Checklist S1 STROBE checklist.
(DOC)

## References

[1] WHO (2012) Dengue and severe dengue. Fact Sheet No. 117. January 2012. Available: http://www.who.int/mediacentre/factsheets/fs117/en/. Accessed 27 January 2013.

[2] Lam SK (1993) Two decades of dengue in Malaysia. Trop Med 35(4): 195–200.

[3] Ministry of Health Malaysia (2010) Clinical practice guidelines on management of dengue infection in adults (revised 2nd ed.). Putrajaya: CPG Secretariat.

[4] Muhammad Azami NA, Salleh SA, Neoh HM, Syed Zakaria SZ, Jamal R (2011) Dengue epidemic in Malaysia: not a predominantly urban disease anymore. BMC Res Notes 4: 216. 21714858 10.1186/1756-0500-4-216 PMC3154160

[5] Cheah WL, Chang MS, Wang YC (2003) Spatial, environmental and entomological risk factors analysis on a rural dengue outbreak in Lundu District in Sarawak, Malaysia. Trop Biomed 23(1): 85–96. 17041556

[6] Fang R, Lo E, Lim TW (1984) The 1982 dengue epidemic in Malaysia: epidemiological, serological and virological aspects. Southeast Asian J Trop Med Public Health 15(1): 51–58. 6740379

[7] Rosenstock IM, Strecher VJ, Becker MH (1994) The health belief model and HIV risk behavior change. In: DiClemente RJ, Peterson JL, editors. Preventing AIDS: theories and methods of behavioral interventions. New York: Plenum Press. pp.5–24.

[8] Janz NK, Champion VL, Strecher VJ (2002) The health belief model. In: Glanz K, Rimer BK, Lewis FM, editors. Health behavior and health education: theory, research, and practice. San Francisco: Jossey-Bass. pp. 45–66.

[9] Phuanukoonnon S, Brough M, Bryan JH (2006) Folk knowledge about dengue mosquitoes and contributions of health belief model in dengue control promotion in Northeast Thailand. Acta Trop 99(1): 6–14. 16945318 10.1016/j.actatropica.2006.05.012

[10] Thompson F, Caltabiano ML (2009) The health belief model and dengue fever preventative behaviours: a pilot programme. Int J Health Promot Educ 48(1): 0–19.

[11] Tsuzuki A, Huynh T, Tsunoda T, Luu L, Kawada H, et al (2009) Effect of existing practices on reducing Aedes aegypti pre-adults in key breeding containers in Ho Chi Minh City, Vietnam. Am J Trop Med Hyg 80(5): 752–757. 19407119

[12] Lennon J (2005) The use of health belief model in dengue health education. Dengue Bull 29: 217–219.

[13] Waltz CF, Strickland O, Lenz E (1991) Measurement in nursing research (2nd ed.). Philadelphia: FA Davis.

[14] Cohen J (1988) Statistical power analysis for the behavioral sciences (2nd ed.). Hillsdale, NJ: Lawrence Erlbaum Associates.

[15] Teixeira Mda G, Barreto ML, Costa Mda C, Ferreira LD, Vasconcelos PF, et al (2002) Dynamics of dengue virus circulation: a silent epidemic in a complex urban area. Trop Med Int Health 7(9): 757–762. 12225506 10.1046/j.1365-3156.2002.00930.x

[16] Ma S, Ooi EE, Goh KT (2008) Socioeconomic determinants of dengue incidence in Singapore. Dengue Bull 32: 17–28.

[17] Pérez-Guerra CL, Zielinski-Gutierrez E, Vargas-Torres D, Clark GG (2009) Community beliefs and practices about dengue in Puerto Rico. Rev Panam Salud Publica 25(3): 218–226. 19454149 10.1590/s1020-49892009000300005

[18] Barreto FR, Teixeira MG, Costa Mda C, Carvalho MS, Barreto ML (2008) Spread pattern of the first dengue epidemic in the city of Salvador, Brazil. BMC Public Health 8: 51. 18257919 10.1186/1471-2458-8-51 PMC2287177

[19] Gubler D (2005) The emergence of epidemic dengue fever and dengue hemorrhagic fever in the Americas: a case of failed public health policy. Rev Panam Salud Publica 17(4): 221–224. 15969972 10.1590/s1020-49892005000400001

[20] Cruz EI, Salazar FV, Aure WE, Torres WP (2008) Aedes survey of selected public hospitals admitting dengue patients in Metro Manila, Philippines. Dengue Bull 32(49): 171–177.

[21] Honorio NA, Nogueira RM, Codeco CT, Carvalho MS, Cruz OG, et al (2009) Spatial evaluation and modeling of dengue seroprevalence and vector density in Rio de Janeiro, Brazil. PLoS Negl Trop Dis 3(11): e545. 19901983 10.1371/journal.pntd.0000545 PMC2768822

[22] Chen CD, Seleena B, Nazni WA, Lee HL, Masri SM, et al (2006) Dengue vectors surveillance in endemic areas in Kuala Lumpur city centre and Selangor state, Malaysia Dengue Bull. 30(17): 197–203.

[23] Wan-Norafikah O, Nazni WA, Noramiza S, Shafa'ar-Ko'ohar S, Azirol-Hisham A, et al (2010) Vertical dispersal of Aedes (Stegomyia) spp. in high-rise apartments in Putrajaya, Malaysia. Trop Biomed 27(3): 662–667. 21399609

[24] Dieng H, Saifur RG, Hassan AA, Salmah MR, Boots M, et al (2010) Indoor-breeding of Aedes albopictus in northern peninsular Malaysia and its potential epidemiological implications. PLoS One 5(7): e11790. 20668543 10.1371/journal.pone.0011790 PMC2910701

[25] Schmidt WP, Suzuki M, Thiem VD, White RG, Tsuzuki A, et al (2011) Population density, water supply, and the risk of dengue fever in Vietnam: cohort study and spatial analysis. PLoS Med 8(8): e1001082. 21918642 10.1371/journal.pmed.1001082 PMC3168879

[26] Win KT, Nang SZ, Min A (2004) Community-based assessment of dengue-related knowledge among caregivers. Dengue Bull 28 28: 189–195.

[27] Young ME, Norman GR, Humphreys KR (2008) Medicine in the popular press: the influence of the media on perceptions of disease. PLoS One, 3(10) : p. e3552. 10.1371/journal.pone.0003552 PMC2569209 18958167

[28] Eisen L, Beaty BJ, Morrison AC, Scott TW (2009) Proactive vector control strategies and improved monitoring and evaluation practices for dengue prevention. J Med Entomol 46: 1245–1255. 19960667 10.1603/033.046.0601

[29] Constantianus JM, Wieteke T, Ratana S, Udom K, Jones J, et al (2006) Dengue knowledge and practices and their impact on Aedes aegypti populations in Kamphaeng Phet, Thailand. Am J Trop Med Hyg 74: 692–700. 16607007

[30] Wan Rozita WM, Yap BW, Veronica S, Mohammad AK, Lim KH, et al (2006) Knowledge, attitude and practice (KAP) survey on dengue fever in an urban Malay residential area in Kuala Lumpur. Malays J Public Health Med 6(2): 62–67.

[31] Hairi F, Ong CH, Suhaimi A, Tsung TW, Anis Ahmad MA, et al (2003) A knowledge, attitude and practices (KAP) study on dengue among selected rural communities in the Kuala Kangsar district. Asia Pac J Public Health 15(1): 37–43. 14620496 10.1177/101053950301500107

[32] Panagos A, Lacy ER, Gubler DJ, MacPherson CNL (2005) Dengue in Grenada. Rev Panam Salud Pública 17: 225–229. 15969973

[33] Pérez-Guerra C, Seda H, García Rivera EJ, Clark G (2005) Knowledge and attitudes in Puerto Rico concerning dengue prevention. Rev Panam Salud Pública 17: 243–253. 15969976 10.1590/s1020-49892005000400005

[34] Quintero A, Carrasquilla G, Suárez R, González C, Olano VA (2009) An ecosystemic approach to evaluating ecological, socioeconomic and group dynamics affecting the prevalence of Aedes aegypti in two Colombian towns. Cad. Saúde Pública, Rio de Janeiro 25(1): S93–S103. 10.1590/s0102-311x2009001300009 19287871

[35] Gubler DJ, Clark GG (1996) Community involvement in the control of Aedes aegypti. Acta Trop 61: 169–179. 8740894 10.1016/0001-706x(95)00103-l

[36] Khun S, Manderson L (2007) Community and school-based health education for dengue control in rural Cambodia: a process evaluation. PLoS Negl Trop Dis 1(3): e143. 18160981 10.1371/journal.pntd.0000143 PMC2154392

[37] Claro LBL, Kawa H, Cavalini LT, Rosa MLG (2006) Community participation in dengue control in Brazil. Dengue Bull 30: 214–222.

[38] Winch P, Leontsini E, Lloyd L (2008) Mosquito control: behavioral and community interventions. In: Halstead SB, editor. Dengue: tropical medicine. Science and Practice Book Series Vol. 5. London: Imperial College Press.

