# Supplementary material for: Community Knowledge, Health Beliefs, Practices and Experiences Related to Dengue Fever and Its Association with IgG Seropositivity
Source: PLoS Negl Trop Dis. 2014 May 22;8(5):e2789. doi: 10.1371/journal.pntd.0002789 (PMC4031145; doi:10.1371/journal.pntd.0002789)
Supplement: Checklist S1 — STROBE checklist. (DOC) [file pntd.0002789.s001.doc]

STROBE Statement—checklist of items that should be included in reports of observational studies

|  | Item No | Recommendation |
| --- | --- | --- |
| **Title and abstract** | 1 | (*a*) Indicate the study’s design with a commonly used term in the title or the abstract  Added in Abstract  A cross-sectional computer-assisted telephone survey of households in a 3km radius of the schools where …. |
|  |  | (*b*) Provide in the abstract an informative and balanced summary of what was done and what was found  Information provided |
| Introduction | | |
| Background/rationale | 2 | Explain the scientific background and rationale for the investigation being reported  Stated in the manuscript |
| Objectives | 3 | State specific objectives, including any prespecified hypotheses  Stated in the manuscript |
| Methods | | |
| Study design | 4 | Present key elements of study design early in the paper  Subsequently, we performed a cross-sectional study in all households….. |
| Setting | 5 | Describe the setting, locations, and relevant dates, including periods of recruitment, exposure, follow-up, and data collection  Described in manuscript |
| Participants | 6 | (*a*) *Cohort study*—Give the eligibility criteria, and the sources and methods of selection of participants. Describe methods of follow-up  *Case-control study*—Give the eligibility criteria, and the sources and methods of case ascertainment and control selection. Give the rationale for the choice of cases and controls  *Cross-sectional study*—Give the eligibility criteria, and the sources and methods of selection of participants  To be eligible for a telephone interview, participants had to be Malaysian, aged between 18 and 60 years old, and resident in the contacted household. Only one person per household was surveyed. If more than one eligible person was found in a household, one person was selected randomly using a random number table. (pg 6) |
|  |  | (*b*) *Cohort study*—For matched studies, give matching criteria and number of exposed and unexposed  *Case-control study*—For matched studies, give matching criteria and the number of controls per case |
| Variables | 7 | Clearly define all outcomes, exposures, predictors, potential confounders, and effect modifiers. Give diagnostic criteria, if applicable  Clearly stated in Pg 9 |
| Data sources/ measurement | 8* | For each variable of interest, give sources of data and details of methods of assessment (measurement). Describe comparability of assessment methods if there is more than one group  Pg 7 |
| Bias | 9 | Describe any efforts to address potential sources of bias  Interviews were conducted after working hours to avoid responses from non-working participants. Random sampling. |
| Study size | 10 | Explain how the study size was arrived at  Sample size not calculated, all the community within 3km radius with landline were contacted. |
| Quantitative variables | 11 | Explain how quantitative variables were handled in the analyses. If applicable, describe which groupings were chosen and why |
| Statistical methods | 12 | (*a*) Describe all statistical methods, including those used to control for confounding |
|  |  | (*b*) Describe any methods used to examine subgroups and interactions |
|  |  | (*c*) Explain how missing data were addressed  Missing data or incomplete responses were not included in the analyses. |
|  |  | (*d*) *Cohort study*—If applicable, explain how loss to follow-up was addressed  *Case-control study*—If applicable, explain how matching of cases and controls was addressed  *Cross-sectional study*—If applicable, describe analytical methods taking account of sampling strategy |
|  |  | (*e*) Describe any sensitivity analyses |

Continued on next page

| Results | | |
| --- | --- | --- |
| Participants | 13* | (a) Report numbers of individuals at each stage of study—eg numbers potentially eligible, examined for eligibility, confirmed eligible, included in the study, completing follow-up, and analysed Figure 1 |
|  |  | (b) Give reasons for non-participation at each stage  The most common reasons for refusal to participate were 'too busy' and 'not interested'. (pg 11) |
|  |  | (c) Consider use of a flow diagram Figure 1 aid the illustration |
| Descriptive data | 14* | (a) Give characteristics of study participants (eg demographic, clinical, social) and information on exposures and potential confounders Summarize in table 1 fist column |
|  |  | (b) Indicate number of participants with missing data for each variable of interest In the table, all the data are complete response, incomplete responses were excluded from the analyses, as indicated in page 11  “ total of 1,400 complete responses were obtained and analysed” |
|  |  | (c) *Cohort study*—Summarise follow-up time (eg, average and total amount) |
| Outcome data | 15* | *Cohort study*—Report numbers of outcome events or summary measures over time |
|  |  | *Case-control study—*Report numbers in each exposure category, or summary measures of exposure |
|  |  | *Cross-sectional study—*Report numbers of outcome events or summary measures (Table 1) |
| Main results | 16 | (*a*) Give unadjusted estimates and, if applicable, confounder-adjusted estimates and their precision (eg, 95% confidence interval). Make clear which confounders were adjusted for and why they were included (Table 1) |
|  |  | (*b*) Report category boundaries when continuous variables were categorized |
|  |  | (*c*) If relevant, consider translating estimates of relative risk into absolute risk for a meaningful time period |
| Other analyses | 17 | Report other analyses done—eg analyses of subgroups and interactions, and sensitivity analyses |
| Discussion | | |
| Key results | 18 | Summarise key results with reference to study objectives |
| Limitations | 19 | Discuss limitations of the study, taking into account sources of potential bias or imprecision. Discuss both direction and magnitude of any potential bias  Pg 22  First and foremost, because individual participant’s seropositivity for dengue-specific IgG was not tested, we cannot conclude that individual respondent’s level of IgG seropositivity was similar to the level of IgG seropositivity of the community nearby the school student population. However, the parallel increase in fogging frequencies along with the increase in the proportion of the community with IgG seropositivity may imply that the seroprevalence of dengue amongst the students that was used as a surrogate of prevalence of dengue in the community in this study may accurately portray the community dengue situation. In Malaysia fogging is most often performed in dengue affected areas. Secondly, a coverage of 3 km radius distance perhaps may not be representative of all community’s children attending the school, and further, dengue may be contracted elsewhere in region though the use of students serology as surrogate indictor of community dengue prevalence was to minimize this potential as students are not likely to have travelled far from their home surrounding and schools. Thirdly, households without a landline telephone were not represented in the study, and further there are a growing number of households with mobile phones and no landline phones. The third limitation of this study is the low response rate of 27.8%, but this is nonetheless very common in telephone surveys. The forth limitation was that although the results may imply health belief model's association with dengue preventive behaviors, the appropriateness of attitudes conceptualized by the HBM in the case of dengue, where repetitive preventive measures are to be performed on a daily basis and, in particular, the outbreak of dengue is seasonal, have raised concerns [38]. Lastly, there is the possibility of bias entailed in general telephone-based surveying, where the data collected were self-reported and may be subject to reporting bias. |
| Interpretation | 20 | Give a cautious overall interpretation of results considering objectives, limitations, multiplicity of analyses, results from similar studies, and other relevant evidence |
| Generalisability | 21 | Discuss the generalisability (external validity) of the study results |
| Other information | | |
| Funding | 22 | Give the source of funding and the role of the funders for the present study and, if applicable, for the original study on which the present article is based |

*Give information separately for cases and controls in case-control studies and, if applicable, for exposed and unexposed groups in cohort and cross-sectional studies.

**Note:** An Explanation and Elaboration article discusses each checklist item and gives methodological background and published examples of transparent reporting. The STROBE checklist is best used in conjunction with this article (freely available on the Web sites of PLoS Medicine at http://www.plosmedicine.org/, Annals of Internal Medicine at http://www.annals.org/, and Epidemiology at http://www.epidem.com/). Information on the STROBE Initiative is available at www.strobe-statement.org.
